# Editorial: The chemo-biological language of plants: exploring the diversity of specialized metabolites

**DOI:** 10.3389/fpls.2023.1226864

**Published:** 2023-06-20

**Authors:** Teresa Docimo, Vincenzo D’Amelia, Anna Lisa Piccinelli

**Affiliations:** ^1^ National Research Council, Institute of Bioscience and Bioresources, Portici, Italy; ^2^ Department of Pharmacy, University of Salerno, Fisciano, Salerno, Italy

**Keywords:** specialized metabolites, invasive species, metabolomics (OMICS), allelopathic effects, biosynthetic pathway analysis, terpenes and phenolics

Plants speak different “languages”. Plant languages are composed of different chemo-biological words differently biosynthesized depending on genetic backgrounds and response to the surrounding environment. The different range of molecules synthetized is also a success of the activity of the photosynthetic apparatus. By harnessing the energy of light, plants are capable to produce primary or “common” precursors that fuel numerous biochemical pathways. A strategy to overcome the limits of a sessile nature. In fact, plants produce a multitude of compounds, often species-specific, which play important ecological roles; substances with different biological properties produced to cope with stresses and which often become nutrients or medicines for humans and animals. Six papers with previously unpublished results on the chemobiological language of plants have been published in this Research Topic. Two of these reported the diversification of chemical profile based on the developmental stage of the plant or the analytical processes used, while the remaining studies unveiled new biosynthetic steps important for the production of metabolites involved in the interaction with the environment.

## Exploration and exploitation of the chemo-biological language

**Figure 1:**
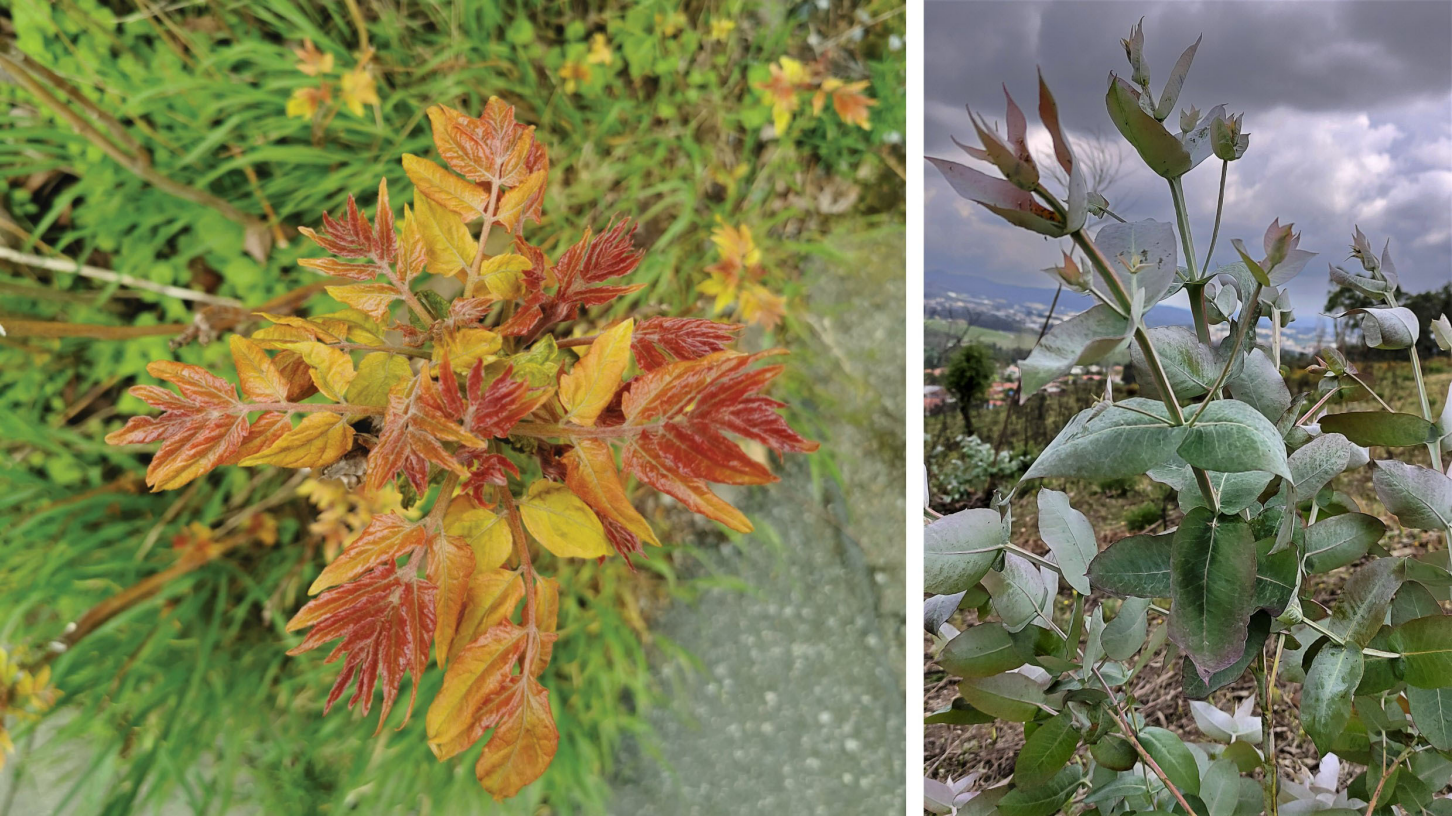
*Alianthus altissima* (*left*) and *Eucalyptus globulus* (*right*) are two invasive species whose exploration of the chemical-biological language opens up new opportunities for human use.

Plants use different molecules to communicate different conditions: alert neighboring plants of pest attack, communicate through positive interaction with organism from other kingdoms, or adopt invasive behavior with other plants to colonize ecological niches (Kliebenstein et al, 2023; Moore et al., 2014). At present, the combination of high-throughput omics techniques is a routine approach that allows the identification of candidate genes that have undergone mutations or duplications, thus extending the potential of plants to produce new molecular complexity. Weeds, invasive species, medicinal herbs or crop wild relatives maintain a large metabolic biodiversity, becoming a fascinating material for exploring the different chemo-biological languages in land plants. Invasive species speak the chemical language of defeat (Hooper et al., 2015). In this Research Topic two manuscripts covered interesting chemical features of *Alianthus altissima* and *Eucaliptus globulus* (**Figure 1**). Not surprisingly, the main compounds exploited by these colonizing plants for a successful invasion belong to compounds derived from benzene, phenols, hydroxamic acids and terpenes (Massalha et al., 2017). Quassinoids are a class of highly modified triterpenoids and represent the allopathic army of *Alianthus altissima* (fam. Simaroubaceae), an invasive species native to China and Nord Vietnam.

Discovering the first three steps of quassinoid biosynthesis, Chuang et al. experimentally confirmed the biochemical relationship of quassinoids with limonoids, another class of triterpenoids produced by plants of sister families Rutaceae and Meliaceae. Melianol was found to be the biosynthetic intermediate that undergoes divergent structural decoration leading to the molecules characterizing the two group of plants. A different chemical solution possibly due to diverse environmental pressures.

The chemo-language interaction between plants can even trigger an intriguing biochemical response that can be exploited for human needs (Khan et al, 2016). *Fritillaria hupehensis* reprograms its metabolism and related gene expression during intercropping with *Magnolia officinalis* (Duan et al.). The interaction between the two plants stimulates *Fritillaria* to arm their bulbs with an increased amount of steroidal alkaloids with an extra energy supply coming from the acceleration of oxidative phosphorylation. Since the latter is a perennial grass of traditional Chinese medicine, this cultivation system is particularly functional in supporting the production of bioactive compounds. The metabolic repertoire can be limited by a single nucleotide mutation in the gene encoding enzymes of key biochemical steps, even in plants of the same genus. This is what Miranda et al. found in *Malus* accessions. Four synonymous mutations in a 3-hydroxylase gene were responsible for the distinctive enzymatic activity leading to the unequal accumulation of dihydrochalcone. The 3-hydroxylation of phloretin leads to the accumulation of sieboldin mainly in wild malus *Malus toringo* and *Malus micromalus* and in some cultivars carrying this “wild allele”. Sieboldin is a powerful antioxidant and, although biosynthetically costly, is very effective in promoting disease resistance. Another dihydrochalcone that confers sweetness is often in co-presence with trilobatin (Gutierrez et al., 2018). The identified DNA variance can be a marker in breeding programs to introgress not only defense traits, but also quality traits given by metabolites. Conversely, metabolites can be biomarkers for predicting complex agronomic traits and, consequently, be used to screen segregating population for agronomic prediction and crop improvement. For example, this is what has been proposed for foxtail millet by Wei et al., who reported that the flavonoid-lignin pathway was correlated and genetically linked with plant architecture and yield. Understanding the chemo-language of plants is challenging but necessary for its exploitation. A targeted use of these plant extracts must always rely on an accurate phytochemical profiling, a pre-requisite for a well-founded use of plant molecules in human activities. Pinto et al. performed a thorough profiling of the allelopathic arms of *E. globulus* from extracts of young and mature leaves, elucidating a different composition in specialized metabolites based on the age of the plant. With such information, the extracts prepared with fresh leaves of young *Eucalyptus* could be more suitable for antioxidant applications in the cosmetic/pharmacological industry while those prepared with dried leaves can be used as eco-friendly herbicides being more enriched in flavonoids or terpenes, respectively. The most appropriate downstream application of the extracts is also influenced by post-harvest processing along with genetic background. This is particularly true when it comes with aromatic plants which must retain the entire bouquet of the aroma. In this Research Topic, this topic has been proposed by Kalalagh et al. who showed the importance of choosing the correct drying temperature to enhance the composition and aroma profile of the essential oils of different dill ecotypes (*Anethum graveolens*).

## Chemodiversity: the useful appeal of plants

In this Research Topic, the chemo-languages of plants has been explored at different levels. While any of the six articles here published dealt with volatile organic compounds, which are very complex mediators in plant communication, novel research perspectives have been proposed. Genomic difference between ecotypes or wild crop relatives, enzymatic capabilities of invasive species, agronomic methods or plant phenology have been shown to impact chemodiversity.

Accurate analytic procedures in the characterization of plant metabolites have also been suggested as the key to proper exploitation. The interplay between increasingly advanced omics technologies has enabled a better understanding of the relationships between genes and metabolites which sheds light on plant metabolic coevolutionary traits or divergent evolution, as well as the unique metabolic signatures representing some plant species. All these tools pave the way for novel biotechnological approaches to exploit the richness of plant chemicals for sustainable uses in a pharmaceutical, agronomic, and industrial perspective.

## Author contributions

TD, VDA, and ALP contributed to the writing of this editorial. All authors revised and improved the final version of the editorial.
